# Competency development for pharmacy: adopting and adapting the FIP global advanced development framework

**DOI:** 10.3389/fmed.2024.1442643

**Published:** 2024-08-14

**Authors:** Asmaa Al-Haqan, Salah Waheedi, Israa Abdullah, Sherly Meilianti, Jenan Shaaban

**Affiliations:** ^1^Department of Pharmacy Practice, College of Pharmacy, Kuwait University, Kuwait City, Kuwait; ^2^International Pharmaceutical Federation, The Hague, Netherlands; ^3^Clinical Pharmacy Unit, Kuwait Hospital, Sabah Al Salem, Kuwait; ^4^Office of Assistant Undersecretary for Medicines and Medical Supplies Affairs, Ministry of Health, Kuwait City, Kuwait

**Keywords:** Kuwait, workforce development and training, mixed methods, research methodology, competency based education, pharmacy, advanced pharmacy practice

## Abstract

**Background:**

Pharmacy education shifts toward competency-based training to meet healthcare demands. This study aims to develop and validate the Kuwait Advanced Competency Framework (KACF) for pharmacists. The study adopts the FIP Global Advanced Development Framework (GADF) to develop a country-specific framework, emphasizing the importance of aligning with global standards while adapting to local contexts. The developed framework builds upon the Kuwait Foundation Competency Framework to address the need for advanced pharmacy services.

**Methods:**

This is a mixed methods study that employed an “adopt and adapt” approach. The KACF was adopted from the FIP GADF and adapted following four phases. Phase one involved checking and validating the Arabic version of the FIP GADF. Phase two employed a series of focus groups to validate accuracy and relevancy of competency statements. Phase three utilized a workshop with different stakeholders as a final step of validation. Phase four involved a national survey to assess the national pharmacy workforce against the framework competencies. Qualitative feedback from focus groups and workshops informed competencies modifications. Quantitative data were analyzed using descriptive and multiple correspondence analyses (MCA).

**Results:**

The translation phase verified a bilingual framework that could be utilized by pharmacists in Kuwait. The initial and final validation phases identified 20 behavioral statements (out of 22 in the original document) that are relevant to pharmacy practice in Kuwait. The national survey, comprising 169 respondents, validated the KACF’s applicability, revealing variations in career stage progression across competency clusters. Findings highlighted associations between career stages and practice settings, offering insights for tailored workforce development strategies.

**Conclusion:**

The KACF emerges as a pivotal tool for advancing pharmacy services in Kuwait, aligning with global trends toward competency-based education. Findings underscored the necessity for context-specific approaches in advancing pharmacy practice, providing a comprehensive understanding of competency progression and readiness for advanced roles.

## Introduction

The changing needs of patients and healthcare systems necessitate that pharmacy workforce education and training goes beyond the traditional emphasis on attaining and applying knowledge, to focusing on learner outcomes and the competencies required for practice (1). In recent years, competency-based education and training (CBET) has been commonly employed for pharmacists (2–4). Competence and competencies are terms becoming increasingly accepted at a global level in healthcare and are being directly linked to professional roles. Competency-based approaches put professional practice at the core of education and practitioner development program. Competency frameworks have become increasingly popular due to the need for transparency in the training, development, and accreditation of healthcare professionals. A competent pharmacy workforce is fundamental for ensuring the provision of high-quality healthcare services. Maintaining competence means a commitment to deliver the full range of pharmaceutical services and to meet the challenges facing global health and patient care.

The International Pharmaceutical Federation (FIP) has created early career maps and developed competency frameworks to support a seamless transition into early career practice and toward advanced practice (5). Moving from the foundation level of practice toward providing more advanced and specialized services dictates the provision of support and guidance. Competency frameworks have been shown to facilitate improvement in pharmacists’ performance (4). In 2020, the FIP presented the Global Advanced Development Framework (GADF), which is a validated tool intended to support the professional development and recognition of the pharmacy workforce everywhere (6). The framework’s primary purpose is to identify broad areas for professional development and advancement so that pharmacists can develop their careers in a structured manner. The GADF was used as a precursor for developing country-specific framework (2).

In Kuwait, pharmacy practice has long been identified as a sector that is yet to be developed. With the announcement of the ‘new Kuwait vision 2035’, planning for pharmacy practice and workforce development and improvement has become imperative. Previous work done in Kuwait has identified the priority goals for pharmacy workforce development, with competency development, early career training, and continuing professional development as the top three priorities for pharmacy workforce development in the country (7). To tackle the first priority several projects were conducting by the College of Pharmacy (CoP) at Kuwait University and the Ministry of Health (MoH). At the undergraduate level the CoP recently transformed its curriculum from a 5-year Bachelor of Pharmacy program to a competency-based 7-year professional doctorate (PharmD). The CoP had successfully developed and implemented an undergraduate competency framework with entrustable professional activities (EPAs) and competency-based assessment methods to support its competency-based education efforts, tailored to meet the needs of Kuwait (8). After graduation and to ensure the continuum of competency-based education, another study was conducted to target the second and third priority and to develop the Kuwait Foundation Competency Framework (KFCF) (9). In 2019, the KFCF was developed (9), and since then, it has been utilized as a developmental tool to support pharmacists’ performance at the foundation level and used as a guide for developing CBET programs for pharmacists in Kuwait. In the same year, the Kuwait Ministry of Health (MoH) established a steering committee that overlooks pharmacy profession expansion and development. In its mandate, the committee was responsible for designing and delivering professional development programs and strategies for the workforce in the pharmaceutical sector. One deliverable to achieve this objective was to develop and validate an advanced competency framework for pharmacists.

Pharmacy services in Kuwait are experiencing a paradigm shift toward advanced practice to meet the evolving healthcare needs of its population. With a growing burden of chronic diseases, an aging population, and increasing demand for specialized care, there is a pressing need to enhance pharmacy services to optimize patient outcomes. Advanced pharmacy services encompass a broad spectrum of clinical activities beyond traditional dispensing, including medication therapy management, therapeutic drug monitoring, and collaborative care with other healthcare providers. These services are integral to promoting rational medication use, improving medication adherence, preventing medication-related complications, and achieving therapeutic goals. Moreover, advanced pharmacy practice aligns with global trends toward expanding the role of pharmacists as essential members of the healthcare team, contributing to interprofessional collaboration and comprehensive patient care. In Kuwait, the implementation of advanced pharmacy services can enhance the efficiency and effectiveness of healthcare delivery, reduce healthcare costs, and ultimately improve patient satisfaction and health outcomes. Therefore, recognizing the importance of advancing pharmacy services is crucial for addressing the complex healthcare needs of Kuwait’s population and ensuring the delivery of high-quality, patient-centered care.

A previous study (9) developed the Kuwait Foundation Competency Framework (KFCF) for pharmacists in order to set the solid groundwork for advancing the pharmacy workforce in Kuwait. This study is a continuation of the development of competency frameworks for pharmaceutical service provision in Kuwait. This study aimed to develop and validate the Kuwait Advanced Competency Framework (KACF) for pharmacists using the FIP GADF with an adopt and adapt approach (2, 9). The KACF would aid in refining and expanding the scope of practice, reforming the pharmacy workforce development, and providing the support required to create an accountable, flexible, and adaptable workforce.

For this study, competencies have been defined as “the knowledge, skills, attitudes and behaviors that an individual develops through education, training, development and experience (10)”. Moreover, advanced practice has been defined as “Advanced Practice is a practice that is so significantly different from that achieved at initial registration that it warrants recognition by professional peers and the public of the expertise of the practitioner and the education, training, and experience from which that capability was derived (11).”

## Methods

**Figure 1:**
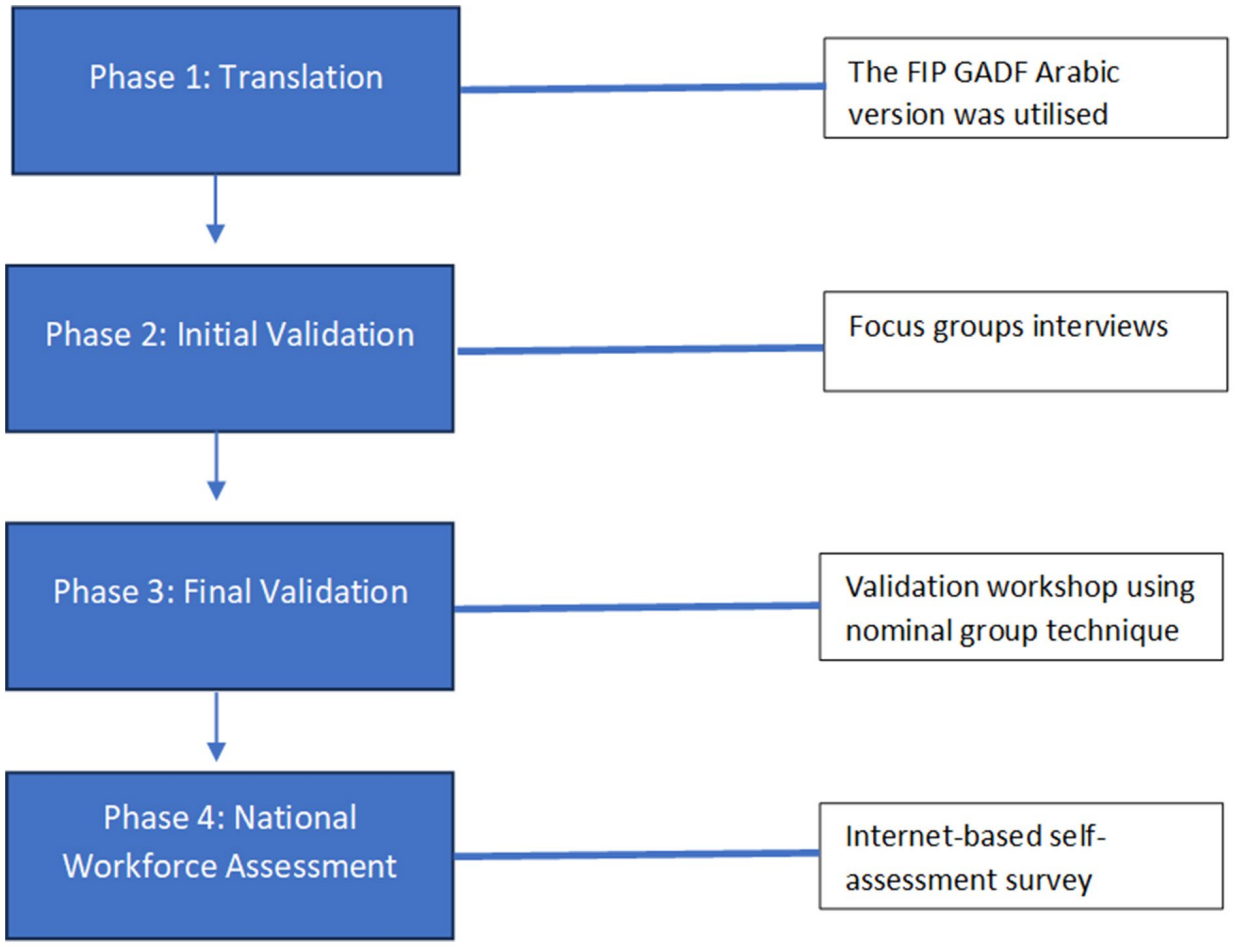
The development process of the Kuwait Advanced Competency Framework (KACF).

This is a mixed methods study that employed an “adopt and adapt” approach similar to Al-Haqan et al. (9) and Meilianti et al. (2). The KACF was adopted from the FIP GADF and adapted to develop a country-specific framework following four phases (Figure 1). The development and validation of the KACF was carried out from February 2022 to January 2023.

### Phase 1: translation

Translation into Arabic was performed to ensure proper utilization of the developed framework by pharmacists in Kuwait, as the official language of the country is Arabic. The FIP GADF was translated into seven languages, including Arabic. Therefore, in this phase, the authors utilized the FIP GADF Arabic version and aimed to validate it throughout other phases. The authors initially checked and validated the Arabic version of the FIP GADF separately. The Arabic translation was then revised by all authors and discussed in several sessions. The revision took into consideration the clarity of the sentences, grammar, wording, and that the meaning, aim, number and order of the sentences match the English phrases. Discrepancies and synonyms were discussed between the authors, and they agreed on a selection of edits. The authors did a second revision following the initial revision through a group meeting to proofread the final draft of the framework.

### Phase 2: initial validation

The initial validation of the study was conducted through qualitative focus group interviews. Three focus group interviews were conducted with two aims: (1) to validate the Arabic translation adopted from the FIP GADF and (2) to investigate the relevancy of the included competencies to Kuwait practice. Three focus groups (*n* = 14) were conducted between February and March 2022. Characteristics of focus group members are presented in Table 1. The focus groups were done online using the Zoom platform. Participants were recruited using purposive sampling according to their practice settings and years in practice and were asked to discuss the following questions: (1) Are competency elements/statements clear and understandable? If not, what modifications/amendments could be made? (2) Is the Arabic translation understandable? If not, what modifications/amendments could be made? (3) Do the competencies belong to the defined cluster? (4) Would you like to add any additional competencies? (5) Do stages (advanced stage 1, advanced stage 2, advanced stage 3) make sense to you? (6) Rate the list of evidence. If not relevant to Kuwait, what do you suggest being included in the list? After each focus group, the research team consolidated all participants’ comments and refined the competencies according to the participants’ suggestions. Comments from one focus group were not presented in the original document but were incorporated into the discussion for other focus groups. After three focus groups, the research team created a draft of the ‘Kuwait Advanced Competency Framework (KACF)’ based on the outcomes of the interviews.

**Table 1:** Characteristics of focus groups participants.

Focus group 1 (*N* = 4)	Focus group 2 (*N* = 5)	Focus group 3 (*N* = 5)
Hospital Pharmacist	Head of departments in healthcare area offices (all health area)	Clinical Pharmacist
Clinical pharmacist		Hospital Pharmacists
Assistant professor		Clinical Pharmacist – Specialized services
Regulatory body - MoH		Primary care Pharmacist
		Community pharmacy

Primary care pharmacists: pharmacists working in primary care centers, usually dealing with mild and stable conditions and focusing on dispensing medications and patient counseling.

Hospital Pharmacist: work in hospitals, dealing with more complex cases and includes pharmaceutical services for the in-patients, outpatient and medication management.

Clinical Pharmacist: clinical pharmacist in general hospital settings including medical wards and/or general medicine.

Clinical pharmacist with specialized services: clinical pharmacist working in a subspeciality setting such as cardiology, nephrology, geriatric, ambulatory care and other specialized settings.

Head of departments in healthcare area offices: leaders of administrative departments from different healthcare regional administrative offices overseeing and coordinating healthcare hospitals and primary care clinics within that region.

Community pharmacy: retail pharmacy in the private sector that primarily focuses on dispensing medications and providing counseling services to patients.

### Phase 3: final validation

In June 2022, a face-to-face workshop was conducted to validate KACF. In this workshop, 20 pharmacists were invited through purposive sampling and a nominal group technique was employed. Participants’ characteristics are presented in Table 2. Participants were divided into six groups, and each group was assigned to a specific cluster to discuss extensively the competencies in that cluster (translation and relevancy). The authors discuss the previous steps taken and the results from the focus groups and highlighted that this is a unified competency framework that can be equally applied across different work settings. The participants were asked to discuss and write their thoughts on each statement included in the cluster assigned to them, as well as its relevancy, applicability, translation to Arabic, and clarity. The last session of the workshop involved discussing each cluster with the comments from each group with all the participants and asking for further feedback, which was also transcribed separately. After the workshop, the research team discussed all changes, modifications and suggestions received from all participants and created a final version of the ‘Kuwait Advanced Competency Framework (KACF)’ based on the workshop outcomes (Supplementary file 1).

**Table 2:** Characteristics of workshop participants.

#	Cluster	Place of work	Years of experience
1	Expert prof.	Hospital	10 years
2	Expert prof.	Hospital	2 years
3	Expert prof.	Community	19 years
4	Expert prof.	Hospital	16 years
1	Working with others	Hospital	24 years
2	Working with others	Hospital	22 years
3	Working with others	Hospital	9 years
4	Working with others	Polyclinic	6 years
1	Leadership	Hospital	20 years
2	Leadership	Central medical stores	17 years
3	Leadership	Polyclinic	24 years
1	Management	Hospital	22 years
2	Management	Hospital	20 years
3	Management	Polyclinic	23 years
1	Education	Kuwait University, COP*	20 years
2	Education	Polyclinic	16 years
3	Education	Hospital	3 years
1	Research	Kuwait University, COP*	20 years
2	Research	Polyclinic	6 years
3	Research	polyclinic	10 years

*COP, College of Pharmacy.

### Phase 4: national workforce assessment—national survey

A cross-sectional internet-based survey was conducted to assess the validity and practicality of the KACF as a self-assessment tool for pharmacists. The survey tool was adopted and adapted from a survey conducted by Albinana (12) and Meilianti et al. (2). The survey had three sections. Section 1 collected demographic data and information pertaining to the current professional practices of pharmacists. Section 2 was self-assessment utilizing the KACF framework. The final section included an open-ended question asking participants to write any comments related to the framework competencies. Due to the lack of updated list of pharmacist’s emails, the survey distribution followed a similar approach of other studies conducted in Kuwait (9, 13, 14). The anonymous link to the web-based questionnaire was disseminated within established Kuwait pharmacy groups on social media platforms such as Facebook and WhatsApp. Pharmacists were additonally encouraged to share the anonymous link with other eligible participants. Subsequent reminders were dispatched at biweekly intervals over a three-month period (at total of 8 reminders) to promote participation.

### Data analysis

Qualitative data from the focus groups and the workshop were discussed extensively by the research team and added to the modified document after each session. Participants from each focus group were able briefed about the comments and corrections suggested by the previous groups and had the chance to add their own comments and suggestions for further analysis.

The quantitative data obtained from the survey were transferred from the online platform to SPSS version 29.0.0. The data were cleaned and coded before the analysis. For determining the combined stage of each cluster applicable to individual respondents (comprising a total of 6 clusters within the framework), the median staging value within the specific cluster was computed. The descriptive analysis was conducted to illustrate the distribution of cluster staging. A multiple correspondence analysis (MCA) was utilized to explore the relationship between cluster staging, overall cluster staging, and participants’ practice sector.

### Ethical consideration

Ethical approval for this study was obtained from the Ministry of Health’s scientific research ethics committee (1786/2021).

## Results

### Phase 1: translation

In this initial phase, the authors discussed some of the concepts that could be misunderstood or not clear to pharmacists in Kuwait. These concepts were discussed in the subsequent phases with no major changes done on the original FIP GADF document.

### Phase 2: focus groups

After incorporating feedback from three focus groups, a series of modifications were made, primarily minor adjustments, including translation revisions for consistency between the English and Arabic versions. The three focus groups had similar comments on the competency framework, with most feedback focusing on the Arabic translation or simplifying the sentences. Within the fourth competency cluster, the title of competency 4.8 was changed from “Strategic Planning” to “Operational Planning.” Furthermore, competency 4.9 was removed, with its content being merged into the remaining competencies within the same cluster. Similarly, in the sixth competency cluster, competency 6.2 was eliminated, and its aspects were integrated into other competencies within that cluster.

Additional minor refinements involved breaking down some lengthy statements into two separate ones for clearer interpretation, mainly within the second cluster, “Working with Others.” The language used in the fifth competency, “Education, Training, and Development” cluster, was also simplified for better comprehension and understanding. Adjustments were made to the competencies in the fourth and sixth clusters to incorporate elements from the deleted competencies, ensuring comprehensive overall competencies.

### Phase 3: final validation

Participants in the workshop suggested several modifications in all clusters. Majority of participants’ comments were related to improving Arabic translation, and adding more clarification to some wording to make them more understandable,

In cluster 1: Expert Professional Practice, participants emphasized the importance of aligning Arabic translations with English statements, particularly regarding terminology such as “core areas” and “applies.” They suggested adjustments to highlight the necessity for advanced pharmacists to not only develop complex programs but also apply them effectively. One participant noted, “*Evidence does not comply with all clusters and subclusters, therefore it’s very hard to define the situation.” Another participant remarked, “The Arabic translation should be clearer.”*

In cluster 2: Working with others, recommendations centered on refining language to promote a more positive reading experience. This included replacing negative terminology with more neutral or positive alternatives and ensuring accurate Arabic translations. For example, Participants in group 2 wrote, “*The table is written in a negative language* [competency statement 2.1]*. It’s not comfortable to read and causes discomfort. We suggest using fewer negative commands and phrases, should not use words like aggression, antagonism, emotive,* etc. *alternatively, use words like challenging situations.”* They also suggested replacing the words “patients and colleagues” with “healthcare providers and recipients” under stage 1.

Moreover, in cluster 3: Leadership, minor adjustments were made to Arabic wordings to maintain consistency with English phrases, reflecting a focus on linguistic accuracy and clarity. One participant remarked, “*I think we need to add more evidence to this cluster such as: Have you been in enough workshops/courses? Develops protocols in emergency cases as leaderships (pandemic corona), special policy for special drug groups,* e.g.*, PPIs and if they worked on any special training programs for pharmacists and updating guidelines.”*

Cluster 4: Management, participants suggested minor rewording and restructuring of competencies, with a specific emphasis on understanding the needs of change rather than the principles of change (competency statement 4.7). A participant mentioned, *“It would be clearer if we can change the sequence of the clusters.”*

For cluster 5: Education, training, and development, participants proposed that education should proceed by “continuous” to imply the importance of continuous education rather than education alone. They suggested adding the term “continuous” throughout the cluster title and definitions. Moreover, minor changes were made to the Arabic wordings to accurately express the meaning of the English phrases. Participants noted, “*Some of the competencies here are hard to achieve, very limited candidates,”* and *“Mentorship needs more definition, like ‘the 3 a’s’ and how to be a good mentor.*”

Finally, in cluster 6: Research and evaluation, recommendations included refining the cluster definition to better express the goal of improving pharmaceutical practice through evidence-based research. Specific language adjustments were proposed to clarify the responsibilities of research project supervisors and to align with professional standards. As with other clusters, Arabic translations were edited to ensure consistency and accuracy. Group 6 recommended adding to the definition of the cluster “Improving the Pharmaceutical Practice and Performance Using Evidence-Based Research” to further express the goal and meaning of this cluster. One participant suggested, “*The evidence is hard to apply here, maybe we can have more examples?*”

Overall, participants provided general comments expressing support for the KACF framework while suggesting improvements, such as providing definitions alongside evidence within each cluster. Concerns were raised regarding the accessibility of evidence for certain competencies and the potential challenges of compliance without incentives for pharmacists.

### Phase 4: national survey

A total of 169 responses were included in the analysis (see Table 3), revealing a diverse distribution across sectors of practice. The majority of participants were affiliated with general public hospitals (42.6%), followed by primary care (25.4%). Other sectors included specialized public hospitals (10.7%), community pharmacies (5.3%), and academia (2.4%), among others. Gender distribution showed 39.6% males while the remaining majority were females (60.4%). In terms of nationality, 62.7% were Kuwaiti, and 37.3% were non-Kuwaiti. The respondents’ average age was approximately 36.78 years, with a standard deviation of 9.200, while their mean years of experience stood at 12.02 years, with a standard deviation of 8.817.

**Table 3:** Demographics of survey participants.

Demographics	Categories	Respondents (%) (*N*: 169)
Sector of practice	Primary care (policlinic)	43 (25.4%)
	General public hospital	72 (42.6%)
	Private hospital	6 (3.6%)
	Specialized public hospital	18 (10.7%)
	Community pharmacy	9 (5.3%)
	Central medical store	4 (2.4%)
	Food and registration department	3 (1.8%)
	Academia	4 (2.4%)
	Private sector	5 (3.0%)
	Others	5 (3.0%)
Gender	Male	67 (39.6%)
	Female	102 (60.4%)
Nationality	Kuwaiti	106 (62.7%)
	Non Kuwaiti	63 (37.3%)
Age	36.78 ± 9.200 (Mean ± Standard Deviation)	
Years of experience	12.02 ± 8.817 (Mean ± Standard Deviation)	

#### Distribution of cluster staging

**Figure 2:**
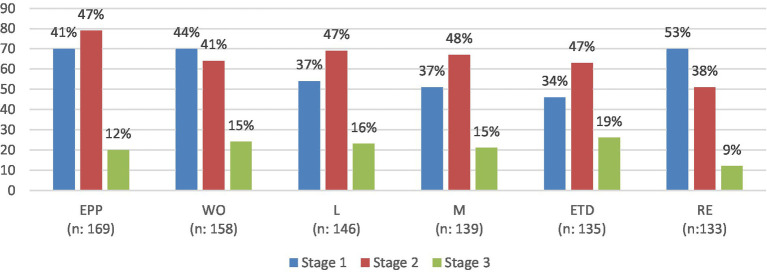
Illustration of the distribution across cluster staging. EPP, Expert professional practice; WO, Working with others; L, Leadership; M, Management; ETD, Education, training and development; RE, research and evaluation.

Figure 2 provides an illustration of the distribution across cluster staging, revealing variations in the progression of expertise across clusters. Four out of six clusters revealed similar distributions where the majority of sample respondents were in stage 2: Expert professional practice, working with others, leadership, management and education and training development. On the other hand, for “working with others” and “research and evaluation” clusters, most respondents were at stage 1, followed by stages 2 and 3. Only 9% of respondents were in stage 3 of the research and evaluation cluster, which showed a lesser readiness of pharmacists for research and evaluation activities.

#### Multiple correspondence analysis (MCA) of KACF mapping

**Figure 3:**
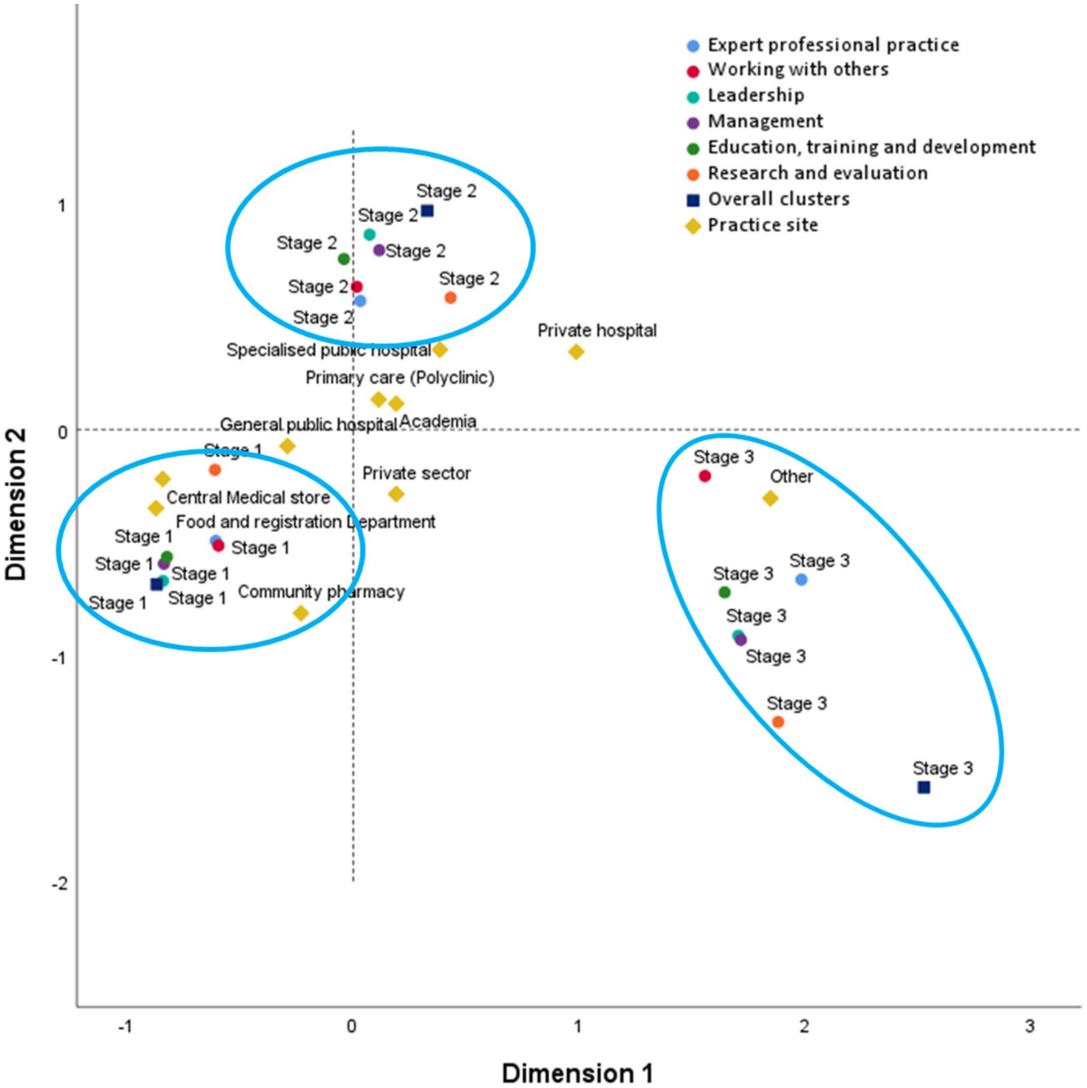
A descriptive visualization of the self-assessed staging and corresponding sectors within the surveyed sample.

Utilizing a two-dimensional MCA model, an exploration of the correlation between cluster staging and the workplace was conducted. The Cronbach’s values computed for both dimensions surpassed the established threshold of 0.5, in accordance with prior research standards, which are 0.894 and 0.780, respectively (15). The analysis summary indicated that this model was robust for association pattern discovery. The joint category plot of the MCA, as depicted in Figure 3, provided a descriptive visualization of the self-assessed staging and corresponding sectors within the surveyed sample. The outcomes of the ‘blue’ groupings highlight the distinct grouping and separation of self-assessed career stages. These results underscore the IADF’s capability, when employed as a self-assessment tool, to effectively differentiate between various stages of career development within this specific sample. The relationship with the practice site showed that the central medical store and food registration department were closely related to stage 1. In contrast, specialized public hospitals, primary care, and academia were closely related to stage 2, and private hospitals were between stage 2 and stage 3.

## Discussion

The study focused on adopting and adapting the FIP GADF to develop a country-specific competency framework. This study employed a series of focus group discussions involving different professional groups and a national survey to assess the pharmacy workforce across cluster staging, illustrating the progression of expertise across clusters. To our knowledge, this framework validation process was the first validation of an advanced competency framework in the Middle East Region. The results revealed several key insights and recommendations for implementation and improvement.

An evolving pharmaceutical workforce is one that can adapt its core roles and responsibilities to meet the new and emerging needs of patients and civil society (5). Competency-based education and training (CBET) in pharmacy aims to equip pharmacists with diverse skills beyond knowledge acquisition to meet evolving patient needs. CBET fosters professionalism, effective communication, and patient-centered care, preparing pharmacists to excel in dynamic healthcare settings and contribute to improved patient outcomes and pharmacy services quality. A study by Koster et al. (16) has demonstrated the effectiveness of competency-based pharmacy education (CBPE) in improving the preparedness of pharmacy graduates for professional practice. Similarly, the work by Austin and Gregory (17) on the Canadian pharmacy practice framework underscores the importance of continuous evaluation and adaptation of competency frameworks to ensure their relevance and efficacy.

In the present study, the FIP GADF was selected as the foundational basis for developing the Kuwait Advanced Competency Framework due to its universal recognition as a benchmark for pharmacy practice and education. Adopting the GADF facilitates alignment with international standards, ensuring that Kuwait’s pharmacists are equipped with globally competitive skills. The subsequent adaptation of the GADF allowed for a nuanced integration of Kuwait’s specific healthcare needs, cultural considerations, and the unique challenges faced by the local pharmacy sector. This careful customization process ensures the framework’s relevance and applicability within Kuwait, significantly enhancing its potential to improve pharmacy practice. Moreover, by outlining key competencies and areas for advancement, the framework supports pharmacists in structuring their professional development and career planning to achieve both local excellence and global competitiveness. For instance, some countries have successfully implemented the GADF, demonstrating its versatility across different healthcare systems (6). Australia and the UK, with their established pharmacy infrastructures, focused on refining advanced practice stages and ensuring the framework addressed both early-career and experienced professionals. Their approach involved extensive stakeholder engagement and iterative modifications to ensure the framework’s relevance and applicability across various pharmacy sectors. In contrast, countries like Indonesia and Jordan adapted the GADF to bolster the foundational competencies of their pharmacists. They concentrated on integrating the framework into existing educational and professional development programs, ensuring that it addressed local practice needs and regulatory requirements.

To meet the unique requirements of Kuwait’s pharmacy sector, we carefully selected participants holding various senior positions for the focus group. Similar to Meilianti et al. (2), the translation process was structured into two different sessions, during which participants were given the opportunity to scrutinize every aspect of the framework in depth. This included a thorough review of the Arabic translation to ensure not only alignment with the original English version but also that the language used was naturally suited for Arabic-speaking pharmacists. This careful approach guaranteed that the translation was both accurate and culturally appropriate, enhancing its accessibility and relevance to the local pharmacy workforce. Other studies done in the region provide a relevant comparison. The work done by Saker et al. (18), and Almaghaslah et al. (19), highlight the importance of customizing these tools to address local needs and contexts. Their experience underscores the value of integrating international standards with local healthcare requirements, mirroring our approach in Kuwait.

In the final phase of our study, 169 pharmacists utilized a newly developed self-assessment framework to determine their competency levels in various areas, providing invaluable feedback on its practicality and effectiveness. Our investigation into cluster staging distribution and its correlation with workplace dynamics shed light on career progression pathways and professional readiness. While our findings align with Meilianti et al. (2) in highlighting prevalent distribution among ‘working with others’ and ‘research and evaluation’ clusters, showing a majority of respondents in Stage 1, a discrepancy emerged. Specifically, our study noted a higher proportion of pharmacists in Stage 2 across four other clusters. Nonetheless, both studies agree on the identified lower readiness among pharmacists.

Differences in competency ratings can stem from various factors, including work context, professional experience, and individual perspectives on competency. Our study observed a modest correlation between workplace settings and staging, suggesting that workplace is associated with how pharmacists self-assessed their career development in Kuwait. For instance, pharmacists working in different settings, such as hospitals, community pharmacies, or academic institutions, may prioritize different competencies based on their specific roles and responsibilities. They may also experience varied opportunities for professional development and skill enhancement, affecting their self-assessed competency levels. Those in hospital settings might report higher competencies in clinical skills, while those in community pharmacies might excel in patient communication and management skills. Professional experience also plays a crucial role in competency assessments. More experienced pharmacists are likely to have developed a broader range of skills and competencies over time, which can influence their self-assessment ratings. Conversely, less experienced pharmacists may identify areas for further development, highlighting the need for targeted training and support.

### Implications for practice

The implications of the study for pharmacy practice are significant and multifaceted. This study yielded a country-specific competency framework and represented a pivotal step toward modernizing pharmacy practice in Kuwait. By aligning with international standards while considering local healthcare needs and cultural nuances, the KACF ensures that pharmacists are equipped with the competencies necessary to excel in their roles and meet the evolving needs of patients and society. The KACF plays a crucial role in preparing pharmacists for contemporary healthcare environments. By prioritizing learner outcomes and practical skills development, KACF empowers pharmacists to navigate complex healthcare landscapes effectively and contribute meaningfully to patient care. The KACF outlines key competencies such as effective communication, professionalism, teamwork, and patient-centered care, reflecting the complex nature of modern pharmacy practice.

The structured approach taken in the development and validation of the KACF ensures its relevance, applicability, and effectiveness within the local pharmacy sector. Through careful selection of participants, meticulous translation processes, and validation through self-assessment frameworks, the KACF has been tailored to meet the unique needs of Kuwait’s pharmacy workforce. The active engagement and support of the pharmacy sector are crucial for the successful adoption and implementation of the KACF, which promises to significantly enhance healthcare quality, address challenges in chronic disease management, and advance pharmacy practice in Kuwait.

Future research should focus on observing the implementation of the KACF within the pharmacy sector and assessing pharmacists’ willingness to adopt it. Longitudinal studies tracking changes in pharmacists’ competency levels over time and evaluating the impact of the KACF on healthcare outcomes will provide valuable insights into its effectiveness and contribution to advancing pharmacy practice in Kuwait. By continuously monitoring and evaluating the framework’s implementation and impact, stakeholders can ensure its ongoing relevance and effectiveness in meeting the evolving needs of patients and society.

### Limitations

While this study benefits from the use of a mixed-methodology approach, allowing for the collection of data through focus groups and a survey to increase the diversity of the data, it is not without limitations. A notable concern is the inherent bias of social desirability present in both the focus groups and the self-administered survey. Participants in focus groups may tailor their responses to align with perceived group norms or to avoid judgment, while individuals completing surveys might similarly report a competency level they believe is more acceptable to their peers. This bias can influence the authenticity of the data collected. Despite efforts to mitigate these biases through careful question design and facilitation techniques, the impact of social desirability cannot be entirely eliminated and represents a limitation to this study.

Another significant limitation concerns the recruitment strategy for survey participants. The study aimed to distribute the web-based survey to all pharmacists (around 4,000 registered pharmacists) in Kuwait via social media and messaging apps, with the hope of achieving wide-reaching participation. However, the response rate of 169 pharmacists suggests potential challenges in dissemination, such as not all pharmacists being reached or aware of the survey. Additionally, it raises the concern that the respondents might predominantly comprise individuals who are particularly enthusiastic or invested in the topic rather than a broad cross-section of the pharmacy workforce. This limitation indicates that the sample may not be representative of the entire pharmacist population in Kuwait, potentially affecting the generalizability of the findings.

## Conclusion

This research sought to establish the Kuwait Advanced Competency Framework (KACF), employing a data-driven methodology to craft precise and meaningful post-graduate education initiatives, aiding in the career development and progression of pharmacists. The KACF is a pivotal tool in modernizing pharmacy practice, aligning international standards with local healthcare needs. Through meticulous development and validation, it equips pharmacists with essential competencies to excel in patient care. Stakeholders’ active support is crucial for successful adoption, ensuring improved healthcare quality and addressing chronic disease challenges. Embracing the KACF signifies commitment to advancing pharmacy practice and enhancing overall health outcomes for Kuwaiti citizens.

## Data availability statement

The raw data supporting the conclusions of this article will be made available by the authors, without undue reservation.

## Ethics statement

Ethical approval for this study was obtained from the Ministry of Health’s scientific research ethics committee (1786/2021). The studies were conducted in accordance with the local legislation and institutional requirements. The participants provided their written informed consent to participate in this study.

## Author contributions

AA-H: Conceptualization, Data curation, Formal analysis, Investigation, Methodology, Supervision, Validation, Writing – original draft, Writing – review & editing. SW: Conceptualization, Data curation, Formal analysis, Investigation, Methodology, Validation, Writing – original draft, Writing – review & editing. IA: Data curation, Formal analysis, Investigation, Methodology, Validation, Writing – original draft, Writing – review & editing. SM: Data curation, Formal analysis, Writing – original draft, Writing – review & editing. JS: Conceptualization, Investigation, Resources, Validation, Writing – review & editing.

## Supplementary material

Supplementary Table 1 Kuwait Advanced Competency Framework for Pharmacists.
