# Supplementary material for: Competency development for pharmacy: adopting and adapting the FIP global advanced development framework
Source: Front Med (Lausanne). 2024 Aug 14;11:1442643. doi: 10.3389/fmed.2024.1442643 (PMC11349709; doi:10.3389/fmed.2024.1442643)
Supplement: Supplementary Table 1 — Kuwait Advanced Competency Framework for Pharmacists. [file Table_1.DOCX]

KUWAIT ADVANCED COMPETENCY FRAMEWORK FOR PHARMACISTS

A framework to support pharmacists to develop their advanced practice and specialisation and assist with their career progression

2022 - Version 1

# Glossary table ^1,2^

| Term | Definition |
| --- | --- |
| Competency | Knowledge, skills, behaviors and attitudes that an individual accumulates, develops, and acquires through education, training, and work experience that is essential to the practice of the profession^3^. |
| Continuing Professional Development | “The responsibility of individual pharmacists for systematic maintenance, development and broadening of knowledge, skills and attitudes, to ensure continuing competence as a professional, throughout their career. ^4^ |
| Core area | Cover the common areas that any pharmacy workforce would be expected to be familiar with in a similar role at an advanced level |
| Defined practice area | The specific area of responsibility in a role, which may be a specialist or generalist that would be covered in depth beyond that of a core area. A defined area may be an area of clinical practice e.g. critical care or a service area e.g. the inpatient pharmacy. |
| Evidence-Based practice | Using good quality evidence to make sound clinical decisions. |
| External environment | Outside of the pharmacy workforce organisation; for example: pharmaceutical services in other organisations, educational institutions, regional or national committees. |
| Governance | A framework through which healthcare organisations are directed and controlled to be accountable for continuously improving the quality of their services and safeguarding high quality of care ^5^. This framework focuses ultimately on delivering safe, effective, and person-centred care to every patient, all of the time ^6^. |
| Higher level | A greater level of organisational complexity than that of the pharmacist’s team. This may include providing expertise and service delivery nationally, regionally, internationally or at a strategic level |
| Resources | Resources can include finances, human, capacity, technology or other resources |
| Service delivery | Relates to the defined area of practice of the individual e.g. health services, educational services, etc |
| Team | A team is a group of professionals working together and mutually accountable for the delivery of a common goal or purpose. Within the context of the framework, “Team” refers to the group (pharmacy or multidisciplinary) with which the pharmacist works most closely and regularly. |
| Working across boundaries | Described as working across professions, sectors or areas of practice |

1. *Adopted from FIP GADF version zero*
2. *Adapted from The RPS Advanced Pharmacy Framework (APF) 2013. The Royal Pharmaceutical Society. Accessed online on 2 September 2013 at http://www.rpharms.com/faculty-documents/rps-advanced-pharmacy-framework-guide.pdf.*
3. *Scope of contemporary pharmacy practice: roles, responsibilities, and functions of pharmacists and pharmacy technicians. J Am Pharm Assoc 2010;50:e35-e69.*
4. *FIP Statement of Professional Standards Continuing Professional Development. Accessed online on May 5 2022 at https://www.fip.org/file/1544*
5. *Healthcare Improvement Scotland. CODE OF CORPORATE GOVERNANCE. Accessed online on May 5 2022 at https://www.healthcareimprovementscotland.org/previous_resources/policy_and_strategy/corporate_governance.aspx*
6. *Macfarlane AJR. What is clinical governance? BJA Educ. 2019;19(6):174-175. doi:10.1016/j.bjae.2019.02.003*

| **Clusters and competencies** | **Stage 1** | **Stage 2** | **Stage 3** |
| --- | --- | --- | --- |
| 1. **Expert Professional Practice**   Improves standards of pharmaceutical care   1. المزاولة المهنية الصيدلانية المبنية على الخبرة   النهوض ورفع مستوى المزاولة المهنية الصيدلانية ضمن القطاع الصيدلاني | | | |
| - 1. Expert Skills and Knowledge   الخبرة والمهارة والمعرفة | Demonstrates **general** pharmaceutical skills and knowledge **in core areas**.  Plans, manages, monitors, advises and reviews programmes in core areas.  أن يظهر الصيدلاني معرفة ومهارة في المجالات الصيدلانية الأساسية العامة. وأن يكون قادرا على تخطيط وإدارة ومتابعة ومراجعة برامج عمل حسب القطاع الصيدلاني. | Demonstrates **in-depth** pharmaceutical skills and knowledge **in defined area**(s).  Plans, manages, monitors, advice and reviews in-depth/complex programmes in defined practice area.  أن يظهر الصيدلاني معرفة عميقة ومهارة متقنة في مجال محدد ضمن القطاع الصيدلاني, وأن يكون قادرا على تخطيط وإدارة ومتابعة ومراجعة برامج عمل عميقة و مركبة في مجال محدد ضمن القطاع الصيدلاني. | **Develops** in-depth/complex programmes in defined practice area.  أن يطور الصيدلاني برامج عمل عميقة/مركبة في مجال محدد ضمن القطاع الصيدلاني. |
| - 1. Developing Professional Expertise, including accountability and responsibility   تطوير الخبرة والمسؤولية المهنية | Demonstrates accountability in providing professional expertise and direct service delivery  أن يتحمل الصيدلاني مسؤولية تقديم خبراته وخدماته المهنية المقدمة بشكل مباشر. | Demonstrates accountability in providing professional services and expertise **via a team** or directly to groups of patients/clients/users.  أن يتحمل الصيدلاني مسؤولية تقديم خبراته وخدماته المهنية ضمن فريق أو بشكل مباشر لمجموعة من المرضى او مستفيدين والمتعامليين | Demonstrates accountability in providing professional expertise at a **defined higher level** (for example nationally, regionally, internationally or at a strategic level).  أن يتحمل الصيدلاني مسؤولية تقديم خبراته المهنية على مستويات عليا محددة (على سبيل المثال: على مستوى محلي, إقليمي, دولي, او استراتيجي). |

| **Clusters and competencies** | **Stage 1** | **Stage 2** | **Stage 3** |
| --- | --- | --- | --- |
| 1. **Expert Professional Practice**   Improves standards of pharmaceutical care   1. المزاولة المهنية الصيدلانية المبنية على الخبرة   النهوض ورفع مستوى المزاولة المهنية الصيدلانية ضمن القطاع الصيدلاني | | | |
| - 1. Reasoning and Judgment   Including: Analytical skills, Judgmental skills, Interpretational skills, Problem solving skills,  Option appraisal  الإستدلال وإصدار الأحكام ويتضمن :  القدرة على التحليل  القدرة على إصدار الإحكام  القدرة على شرح وتفسير المعلومات والحقائق, القدرة على حل المشكلات  القدرة على التقييم والمفاضلة بين الخيارات المطروحة والبدائل المتاحة | Demonstrates ability to use skills in a range of routine situations requiring analysis or comparison of a range of options.  Recognises priorities when problem-solving and identifies deviations from the normal pattern.  أن يكون الصيدلاني قادراً على إستخدام مهارات التحليل وإصدار الأحكام والمفاضلة بين الخيارات في الظروف الروتينية.  وأن يكون الصيدلاني قادراً على تحديد الأولويات عند حل المشكلات والتعرف على الحالات الإستثنائية الخارجة عن المألوف. | Demonstrates ability to use skills to **make decisions** in complex situations where there are several factors that require analysis, interpretation, and comparison.  Demonstrates an ability to see situations holistically.  أن يكون الصيدلاني قادراً على اتخاذ القرار في المواقف المعقدة وذلك عند وجود عوامل تحتاج الى التحليل والشرح والمقارنة  القدرة على رؤية المواقف بأسلوب متكامل وشمولي. | Demonstrates ability to use skills to **manage difficult and dynamic situations**.  Demonstrates ability to make decisions in the absence of established practice, protocols, evidence or data or when there is conflicting evidence or data.  أن يكون الصيدلاني قادراً على إستخدام مهارات لادراة المواقف الصعبة والمتغيرة.  وأن يكون الصيدلاني قادراً على إتخاذ القرارات في حالة غياب الأدلة والمعلومات الكافية أو تضاربها وتعارضها. |
| - 1. Professional Autonomy   الإستقلالية المهنية | Is able to follow legal, ethical, professional and organizational policies/procedures and codes of conduct.  أن يكون الصيدلاني قادراً على إتباع قوانين وأخلاقيات المهنة والسياسات والاجراءات المؤسسية وقواعد السلوك المهني | Is able to take action based on **own interpretation** of broad professional policies/procedures where necessary.  أن يكون الصيدلاني قادراً على التصرف بناء على فهمه وتفسيره للاجراءات و السياسات العامة عند الضرورة. | Is able to interpret relevant policy and strategy, in order to establish goals and standards **for others** within the defined area(s).  أن يكون الصيدلاني قادراً على تفسير وفهم السياسات والإستراتيجيات المرتبطة بمجال محدد لوضع أهداف ومعايير للاخرين |

| 1. **Expert Professional practice**   (See the page above) | **Evidence** | | | | | | | | | | | |
| --- | --- | --- | --- | --- | --- | --- | --- | --- | --- | --- | --- | --- |
|  | Member of, or provide  advice to, a local group or committee | Member of, or provide  advice to, a regional,  national or international  group or committee | Active Teaching role | Educational development,  design & provision | Active research  participation (includes  publications) | Research development & leadership (also includes publications) | Professional standing &  peer status | Documented expert  practice | Managing process &  leadership (local level) | Managing process &  Leadership (national  level) | Staff management | Any OTHER appropriate  Documentation |
| - 1. **Expert Skills and Knowledge**   الخبرة والمهارة والمعرفة  Stage 1 € Stage 2 € Stage 3 € | € | € | € | € | € | € | € | € | € | € | € | € |
| - 1. **Developing Professional Expertise**   تطوير الخبرة والمسؤولية المهنية  Stage 1 € Stage 2 € Stage 3 € | € | € | € | € | € | € | € | € | € | € | € | € |
| **1.3 Reasoning and Judgment**  الإستدلال وإصدار الأحكام  Stage 1 € Stage 2 € Stage 3 € | € | € | € | € | € | € | € | € | € | € | € | € |
| **1.4 Professional Autonomy**  الإستقلالية والمسؤولية المهني  Stage 1 € Stage 2 € Stage 3 € | € | € | € | € | € | € | € | € | € | € | € | € |

| **If any of the competencies was not achieved, explain why** |  |
| --- | --- |

| **Clusters and competencies** | **Stage 1** | **Stage 2** | **Stage 3** |
| --- | --- | --- | --- |
| 1. **Working with Others**   Is able to communicate, establish and maintain professionally driven working relationships and gain the co-operation of others   1. **العمل المشترك والتعاون المهني**   القدرة على التواصل وإنشاء وإدامة علاقات عمل وتعاون مهني مع غيره من الصيدلة وغيرهم من المتعامليين مع الصيادلة والمستفيدين من خدماتهم | | | |
| - 1. Communication   Including ability to:  Persuade, Motivate, Negotiate, Empathise, provide reassurance, Listen, Influence and Empower (includes networking skills and presentation skills)  التواصل والإتصال  ويتضمن ذلك القدرة على الإقناع , والتحفيز, و التفاوض, وتفهم الاّخرين والتعاطف معهم, و طمأنتهم والإستماع والتأثير والتمكين. بالإضافة إلى مهارات بناء علاقات عمل ناجحة ومهارات التقديم والشرح | Demonstrates use of appropriate communication to gain the co-operation of relevant stakeholders (including patients, colleagues, and other professions), and being able to communicate where the content of the discussions is explicitly defined.    أن يكون الصيدلاني قادراً على التواصل بشكل فعال وكسب تعاون المعنيين والمستفيدين من العمل الصيدلاني (مثل المرضى, صيادلة اّخرين, وغيرهم من مختصي الرعاية الصحية) وقادرا على التواصل عند النقاش في مواضيع محددة | Demonstrates use of appropriate communication skills to gain co-operation of **small groups** of relevant stakeholders **within the organization,** and being able to communicate where the content of the discussion is based on **professional opinion**.  أن يظهر الصيدلاني قدرةً على التواصل والنقاش بناء على رأيه المهني والعلمي مع مجموعات صغيرة من المعنيين والمستفيدين من العمل الصيدلاني ضمن المؤسسة الصيدلانية أو مكان العمل. | Demonstrates ability to present complex, sensitive, or contentious information to **large groups** of relevant stakeholders, and being able to communicate in a **hostile, antagonistic or highly emotive atmosphere**.  أن يظهر الصيدلاني قدرةً على إيصال ومناقشة مواضيع ومعلومات معقدة وحساسة ومثيرة للجدل لمجموعات كبيرة من المعنيين والمستفيدين من العمل الصيدلاني، والقدرة على التواصل في حالات المناقشات الحادة والانفعالية . |
| - 1. Teamwork and Consultation   العمل بروح الفريق والتشاور | Demonstrates ability to work as a member of a team, and recognizes personal limitations and refers to more appropriate colleagues.  أن يكون الصيدلاني قادرا على العمل كعضو ضمن الفريق. وأن يكون قادراً على تحديد مواطن ونقاط الضعف لديه وطلب المشورة والعون من زملائه | Demonstrates ability to work as an acknowledged member of a multidisciplinary team, and accepts expert advice through consultation from within the organization.  أن يكون الصيدلاني قادرا على العمل كعضو فعال ضمن فريق عمل من مختلف التخصصات. وأن يتقبل المشورة المهنية من ذوي الاختصاص ضمن مؤسسة العمل. | Works across boundaries to build relationships and share information, plans and resources, and sought as opinion leader both within the organization and in the external environment.  أن يبني الصيدلاني علاقات عمل ويتشارك المعلومات والمعطيات وخطط العمل والموارد. وأن يكون الصيدلاني ذو رأياً مؤثراً ومرجعاً يأخذ بفكره ورأيه داخل وخارج مؤسسة العمل |

| 1. **Working with Others**   (See the page above) | **Evidence** | | | | | | | | | | | |
| --- | --- | --- | --- | --- | --- | --- | --- | --- | --- | --- | --- | --- |
|  | Member of, or provide  advice to, a local or Trust  group or committee | Member of, or provide  advice to, a regional,  national or international  group or committee | Active Teaching role | Educational development,  design & provision | Active research  participation (includes  publications) | Research development &  leadership (also includes  publications) | Professional standing &  peer status | Documented expert  practice | Managing process &  leadership (local level) | Managing process &  Leadership (national  level) | Staff management | Any OTHER appropriate  Documentation |
| - 1. **Communication**   التواصل والإتصال  Stage 1 € Stage 2 € Stage 3 € | € | € | € | € | € | € | € | € | € | € | € | € |
| - 1. **Teamwork and Consultation**   عمل بروح الفريق والتشاور الجماعي تطوير الخبرة والمسؤولية المهنية  Stage 1 € Stage 2 € Stage 3 € | € | € | € | € | € | € | € | € | € | € | € | € |

| **If any of the competencies was not achieved, explain why** |  |
| --- | --- |

| **Clusters and competencies** | **Stage 1** | **Stage 2** | **Stage 3** |
| --- | --- | --- | --- |
| 1. **Leadership**   Inspires individuals and teams to achieve high standards of performance and personal development   1. السمات والمهارات القيادية   القدرة على تشجيع وإلهام وتحفيز الأفراد لتحقيق أعلى مستويات الأداء الوظيفي وتطوير وتنمية وتطوير الذات | | | |
| 3.1. Strategic Context  الإطار الإستراتيجي للمهنة | Demonstrates understanding of the needs of stakeholders and **practice** in a way that reflects relevant local, national, regional or global policy.  أن يزاول الصيدلاني المهنة بناءً على فهمه لإحتياجات المعنيين والمستفيدين من العمل الصيدلاني, وأن تكون مزاولته متماشية ومتناغمة مع السياسات المحلية والوطنية ذات الصلة. | Demonstrates ability to incorporate relevant local, national, regional or global policy to **influence local strategy**.  ان يكون الصيدلاني قادرا على تنفيذ ودمج السياسات المحلية، الاقليمية،او العالمية **للتأثير على الاستراتيجيات المحلية** | Demonstrates active participation in **creating relevant** local, national, regional or global policies.  أن يشارك الصيدلاني بشكل فعال في استحداث **السياسات المحلية** ، والوطنية والإقليمية والعالمية ذات الصلة بالعمل والقطاع الصيدلاني. |
| - 1. Governance (Standards, Quality, and Accountability)   منظومة الأسس والقواعد والمسؤولية المهنية | Demonstrates understanding of the pharmacy role in governance, and able to implement this appropriately within the workplace.  أن يظهر الصيدلاني فهماً للقيم والقواعد والمعايير الناظمة لعمله من حيث الجودة والنوعية ويكون قادراً على تطبيقها والإلتزام بها. | Influences the planning or development of governance processes, for the team and/or service delivery.  أن يكون الصيدلاني ذو تأثير على التخطيط او التطوير للقيم والقواعد والمعايير الناظمة ضمن فريق العمل أو/و الخدمة المقدمة. | Shapes and contributes to the planning or development of governance processes at a **higher level.**  أن يقوم الصيدلاني بوضع وتشكيل القيم والقواعد والمعايير الناظمة للصيدلة على المستويات العليا للمهنة الصيدلانية. |
| - 1. Vision   الرؤية المستقبلية | Demonstrates understanding of, and contributes to, the organization vision.  أن يظهر الصيدلاني قدرةً على فهم الرؤية والتوجه العام لمؤسسة العمل والمشاركة فيها. | **Creates vision** of future and translates this into clear directions for others.  أن يكون الصيدلاني قادراً على تشكيل رؤية مستقبلية وترجمتها لتوجيهات واضحة للاّخرين. | Convinces others to share the vision at a **higher level**.  أن يكون الصيدلاني قادراً على إقناع الآخرين بالرؤية المستقبلية ومشاركتها على المستويات العليا للمهنة الصيدلانية. |
| - 1. Innovation   الإبداع والإبتكار | Demonstrates ability to improve quality w**ithin limitations of service**.  أن يظهر الصيدلاني قدرةً على تحسين الجودة بالرغم من وجود معوقات للخدمة | Recognises and **implements** innovation **from the external environment**.  أن يكون الصيدلاني قادراً على التعرف على الأفكار الإبداعية من المحيط الخارجي لنطاق العمل وتطبيقها داخل نطاق العمل. | **Takes the lead t**o ensure innovation produces **demonstratable improvement in service delivery**.  أن يأخذ الصيدلاني زمام المبادرة لضمان أن يؤدي الابتكار إلى تحسين ظاهر في تقديم الخدمات |

| **Clusters and competencies** | **Stage 1** | **Stage 2** | **Stage 3** |
| --- | --- | --- | --- |
| 1. **Leadership**   Inspires individuals and teams to achieve high standards of performance and personal development   1. السمات والمهارات القيادية   القدرة على تشجيع وإلهام وتحفيز الأفراد لتحقيق أعلى مستويات الأداء الوظيفي وتطوير وتنمية وتطوير الذات | | | |
| - 1. Service Development   تطوير الخدمة | **Reviews** the service progress and develops clear plans to achieve results within priorities set by others.  أن يراجع الصيدلاني سير تقديم الخدمة وأن يضع خطة واضحة لتحقيق النتائج المتوقعة وفقا لأولويات محددة مسبقاً من قبل الاّخرين | Develops clear understanding of priorities and **formulates** practical short-term plans in line with workplace strategy.  أن يكون الصيدلاني قادراً على وضع خطط عمل قصيرة المدى متماشية مع الإستراتيجية العامة بناء على فهمه الواضح لأولويات مكان العمل | Relates service goals and actions to **strategic aim**s of organization and profession.  أن يكون الصيدلاني قادراً على أن يربط أهداف الخدمة مع إستراتيجية المؤسسة والقطاع الصيدلاني بشكل عام. |
| - 1. Motivation   التشجيع والتحفيز | Demonstrates ability to **motivate self** to achieve goals.  أن يظهر الصيدلاني القدرة على تشجيع وتحفيز النفس لتحقيق أهداف العمل. | Demonstrates ability to **motivate individuals** and/or the team.  أن يظهر الصيدلاني قدرةً على تحفيز وتشجيع الأفراد المحيطين به أو/و فريق العمل | Demonstrates ability to motivate individuals and/or teams at a **higher level**.  أن يظهر الصيدلاني قدرةً على تشجيع وتحفيز الأفراد أو\والفرق ضمن المستويات العليا للمهنة الصيدلانية. |

| 1. **Leadership**   (See the page above) | **Evidence** | | | | | | | | | | | |
| --- | --- | --- | --- | --- | --- | --- | --- | --- | --- | --- | --- | --- |
|  | Member of, or provide  advice to, a local or  group or committee | Member of, or provide  advice to, a regional,  national or international  group or committee | Active Teaching role | Educational development,  design & provision | Active research  participation (includes  publications) | Research development & leadership (also includes publications) | Professional standing &  peer status | Documented expert  practice | Managing process &  leadership (local level) | Managing process &  Leadership (national  level) | Staff management | Any OTHER appropriate  Documentation |
| **3.1. Strategic Context**  الإطار الإستراتيجي للمهنة  Stage 1 € Stage 2 € Stage 3 € | € | € | € | € | € | € | € | € | € | € | € | € |
| - 1. **Governance (Standards, Quality, and Accountability)**   منظومة الأسس والقواعد المهنية الحاكمة تطوير الخبرة والمسؤولية المهنية  Stage 1 € Stage 2 € Stage 3 € | € | € | € | € | € | € | € | € | € | € | € | € |
| - 1. **Vision**   الرؤية المستقبلية  Stage 1 € Stage 2 € Stage 3 € | € | € | € | € | € | € | € | € | € | € | € | € |
| - 1. **Innovation**   الإبداع والإبتكار  Stage 1 € Stage 2 € Stage 3 € | € | € | € | € | € | € | € | € | € | € | € | € |
| - 1. **Service Development**   تطوير الخدمة وأساليب العمل الصيدلاني  Stage 1 € Stage 2 € Stage 3 € | € | € | € | € | € | € | € | € | € | € | € | € |
| - 1. **Motivational**   الدافعية والتشجيع والتحفيز  Stage 1 € Stage 2 € Stage 3 € | € | € | € | € | € | € | € | € | € | € | € | € |

| **If any of the competencies was not achieved, explain why** |  |
| --- | --- |

| **Clusters and competencies** | **Stage 1** | **Stage 2** | **Stage 3** |
| --- | --- | --- | --- |
| 1. **Management**   Organizes and delivers service objectives in a timely manner   1. **الإدارة**   *القدرة على إدارة العمل وإنجاز أهدافه بكفاءه وفاعلية* | | | |
| 4.1. Responding and adapting to national needs  الاستجابة والتكيف مع الاحتياجات الوطنية | Demonstrates understanding of the implications of national priorities for the team and/or organization.  أن يظهر الصيدلاني فهماً للأولويات الوطنية في قطاع الصحة وإنعكاسها على مجريات عمله ضمن مؤسسة العمل أو/و ضمن فريق العمل. | Shapes the response of the team and/or organization to national priorities.  أن يكون الصيدلاني قادراً على تشكيل استجابة فريق العمل أو/و مؤسسة العمل للأولويات الوطنية. | Accountable for the direct delivery of national priorities at a **higher level.**  أن يتحمل الصيدلاني مسؤولية التحقيق المباشر للأولويات الوطنية على المستويات العليا |
| - 1. Resource Utilisation   استخدام الموارد | Demonstrates **understanding** of the process for effective resource utilization.  أن يظهر الصيدلاني القدرة على فهم أساليب الإدارة الفعالة للموارد. | Demonstrates ability to effectively **manage** resources.  أن يظهر الصيدلاني قدرةً على إدارة الموارد بفعالية. | Demonstrates ability to **reconfigure** the use of available resources.  أن يكون الصيدلاني قادراً على إعادة توجيه وإستخدام الموارد المتاحة. |
| - 1. Standards of Practice   معايير مزاولة المهنة | Demonstrates understanding of the process of, and conforms to, relevant standards of practice.  أن يظهر الصيدلاني إدراكاً وفهماً وإلتزاماً بمعايير مزاولة المهنة. | Develops and monitors standards of practice at **team level.**  أن يقوم الصيدلاني بوضع ومتابعة معايير مزاولة المهنة لفريق العمل. | Develops and monitors standards of practice at a **higher level**.  أن يقوم الصيدلاني بوضع ومتابعة معايير مزاولة المهنة على المستويات العليا لمهنة الصيدلة. |
| - 1. Managing Risk   **إدارة المخاطر** | Demonstrates ability to identify and resolve risk management issues according to policy/protocol.  أن يظهر الصيدلاني قدرةً على تحديد ومعالجة قضايا إدارة المخاطر وفقاً لسياسات وبرامج عمل محددة مسبقاً. | Develops risk management policies/protocols **for the team and/or organization**, including identifying and resolving new risk management issues.  أن يقوم الصيدلاني بتطوير سياسات وبرامج عمل لإدارة المخاطر **لفريق العمل أو/و مؤسسة العمل** بما في ذلك القدرة على تحديد ومعالجة قضايا إدارة المخاطر المستجدة. | Develops risk management policies/procedures at a **higher level**, including identifying and resolving new risk management issues.  أن يقوم الصيدلاني بتطوير سياسات وبرامج عمل لإدارة المخاطر بما في ذلك القدرة على تحديد ومعالجة قضايا إدارة المخاطر المستجدة على ا**لمستويات العليا لمهنة الصيدلة.** |

| **Clusters and competencies** | **Advanced Stage 1** | **Advanced Stage 2** | **Advanced Stage 3** |
| --- | --- | --- | --- |
| 1. **Management**   Organizes and delivers service objectives in a timely manner   1. الإدارة   *القدرة على إدارة العمل وإنجاز أهدافه بكفاءه وفاعلية* | | | |
| - 1. Managing Performance   إدارة ومتابعة الأداء الوظيفي | Follows professional and organizational policies /procedures relating to performance management.  أن يكون الصيدلاني ملتزماً ويتبع قواعد ومعايير الأداء الوظيفي لمهنة الصيدلة ولمؤسسة العمل. | Contributes to performance management for **a team**.  أن يساهم الصيدلاني بفاعلية في إدارة ومتابعة الأداء الوظيفي لفريق العمل. | Contributes to the performance management at **a higher level**.  أن يساهم الصيدلاني بفاعلية في إدارة ومتابعة الأداء الوظيفي على المستويات العليا لمهنة الصيدلة. |
| - 1. Project Management   إدارة المشاريع | Demonstrates understanding of the principles of project management.  أن يظهر الصيدلاني فهماً لمبادئ إدارة المشاريع. | Demonstrates ability to successfully manage a project at **team and/or organization level**.  أن يظهر الصيدلاني قدرةً على إدارة المشاريع بنجاح على مستوى فريق العمل أو/و مؤسسة العمل. | Demonstrates ability to successfully manage a project at **a higher level**.  أن يظهر الصيدلاني قدرةً على إدارة المشاريع بنجاح على المستويات العليا لمهنة الصيدلة. |
| - 1. Managing Change   إدارة التغيير | Demonstrates understanding of the principles of change management.  أن يظهر الصيدلاني فهماً لمبادئ إدارة التغيير. | Demonstrates ability to manage a process of change for **the team and/or organization**.  أن يظهر الصيدلاني قدرةً على إدارة التغيير على مستوى فريق العمل أو/و مؤسسة العمل. | Demonstrates ability to **promote, initiate and/or lead** a process of change at a **higher level**.  أن يظهر الصيدلاني قدرةً على المبادرة بعملية التغيير والترويج لها و\أو قيادتها على المستويات العليا لمهنة الصيدلة. |
| - 1. Operational Planning   **ا**لتخطيط التشغيلي | Demonstrates ability to plan and deliver the desired outcomes according to the proposed strategy.  أن يكون الصيدلاني قادراً على التخطيط وتحقيق النتائج المرجوة بناءً على الخطة الإستراتيجية المتبناه في مؤسسة العمل. | Demonstrates ability to plan and deliver the desired outcomes, while **adapting the planning** and strategy based on the changes in internal and external environment.  أن يكون الصيدلاني قادراً على التخطيط وتحقيق النتائج المرجوة اّخذاً بعين الإعتبار التغيرات في مناخ وبيئة والعوامل الداخلية والخارجية المؤثرة على سير ونهج العمل وإنعكاسها على خططه وسياساته. | Demonstrates **long term** **and\or sector wide** strategic planning and understanding of organizational politics changes in the external environment.  أن يظهر الصيدلاني قدرةً على وضع خطط إستراتيجية طويلة المدى وشاملة لمختلف المجالات ضمن القطاع الصيدلاني, وأن يكون الصيدلاني واعياً ومدركاً للتغيرات والعوامل الخارجية التي تؤثر على سياسات مؤسسة العمل. |

| **4. Management**  (See the page above) | **Evidence** | | | | | | | | | | | |
| --- | --- | --- | --- | --- | --- | --- | --- | --- | --- | --- | --- | --- |
|  | Member of, or provide  advice to, a local or Trust  group or committee | Member of, or provide  advice to, a regional,  national or international  group or committee | Active Teaching role | Educational development,  design & provision | Active research  participation (includes  publications) | Research development &  leadership (also includes  publications) | Professional standing &  peer status | Documented expert  practice | Managing process &  leadership (local level) | Managing process &  Leadership (national  level) | Staff management | Any OTHER appropriate  Documentation |
| 4.1**. Responding and adapting to national needs**  تطبيق الأولويات الوطنية المتعلقة بالقطاع الصحي والصيدلاني  Stage 1 € Stage 2 € Stage 3 € | € | € | € | € | € | € | € | € | € | € | € | € |
| - 1. **Resource Utilisation**   استخدام وتسخير الموارد والمصادر  Stage 1 € Stage 2 € Stage 3 € | € | € | € | € | € | € | € | € | € | € | € | € |
| - 1. **Standards of Practice**   معايير الأداء الوظيفي حكام  Stage 1 € Stage 2 € Stage 3 € | € | € | € | € | € | € | € | € | € | € | € | € |
| - 1. **Managing Risk**   إدارة المخاطر  Stage 1 € Stage 2 € Stage 3 € | € | € | € | € | € | € | € | € | € | € | € | € |
| - 1. **Managing Performance**   إدارة ومتابعة الأداء الوظيفي  Stage 1 € Stage 2 € Stage 3 € | € | € | € | € | € | € | € | € | € | € | € | € |
| - 1. **Project Management**   إدارة المشاريع والبرامج  Stage 1 € Stage 2 € Stage 3 € | € | € | € | € | € | € | € | € | € | € | € | € |
| - 1. **Managing Change**   إدارة التغيير والتطوير التنظيمي  Stage 1 € Stage 2 € Stage 3 € | € | € | € | € | € | € | € | € | € | € | € | € |
| - 1. **Operational Planning**   **ا**لتخطيط الإستراتيجي  Stage 1 € Stage 2 € Stage 3 € | € | € | € | € | € | € | € | € | € | € | € | € |

| **If any of the competencies was not achieved, explain why** |  |
| --- | --- |

| **Clusters and competencies** | **Stage 1** | **Stage 2** | **Stage 3** |
| --- | --- | --- | --- |
| 1. **Education, Training, and Development**   Supports the education, training, and development of self and others. Promotes a learning culture within the organization.   1. التعليم والتدريب والتطوير المستمر   *القدرة على دعم تعليم وتدريب و تطور الاّخرين ونشر ثقافة التعلم والتطوير المستمر ضمن مؤسسة العمل.* | | | |
| 5.1. Role Model  القدوة | Understands and demonstrates the characteristics of a role model to members in the **team and/or organization.**  أن يكون الصيدلاني مدركاً ومظهراً لسمات القدوة لزملائه ضمن فريق العمل أو/و مؤسسة العمل. | Demonstrates the characteristics of an effective role model at a **higher level.**  أن يكون الصيدلاني مظهرا لسمات القدوة بشكل فعال على المستويات العليا لمهنة الصيدلة. | Is able to **develop** effective role model behavior **in others**.  أن يكون الصيدلاني قادراً على تطوير سمات القدوة الفعالة لدى الاّخرين. |
| - 1. Mentorship   الإرشاد والتوجيه | Demonstrates **understanding** of the mentorship process.  أن يظهر الصيدلاني فهما لعملية التوجيه والإرشاد. | Demonstrates ability to effectively mentor others **within** **the** **team**.  أن يظهر الصيدلاني القدرة على توجيه وإرشاد الاّخرين بشكل فعال ضمن فريق العمل | Demonstrates ability to effectively mentor **outside the team.**  أن يظهر الصيدلاني القدرة على توجيه وإرشاد الاّخرين خارج حدود فريق العمل. |
| - 1. Conducting, Education and Training   التعليم والتدريب | Demonstrates ability to deliver teaching and feedback effectively according to a learning plan  أن يكون الصيدلاني قادراً على أن يعلم غيره ويقيمهم بشكل فعال بناء على خطة تدريب | Demonstrates ability to **evaluate** the learning performance and learning needs of others and **plan** a series of effective learning experiences for them.  أن يكون الصيدلاني قادراً على تقييم أداء الاّخرين وتحديد حاجاتهم التدريبة والتعليمية, وأن يشارك في وضع برنامج تعليمي فعال ومناسب لهم. | Demonstrates ability to **design** and **manage** a course of study, with appropriate use of teaching, learning and study methods.  أن يكون الصيدلاني قادراً على وضع وإدارة برنامج تدريبي بإستخدام وسائل التعليم والتدريب المناسبة. |
| - 1. Professional Development   التطوير المهني | Demonstrates self-development through professional development activity.  أن يظهر الصيدلاني قدرةً على تطوير نفسه من خلال نشاطات التطوير المهني. | Facilitates the professional development of others.  أن يعمل الصيدلاني على مساعدة الاّخرين ودعمهم في تطورهم المهني. | Shapes and contributes to the professional development strategy.  أن يشارك الصيدلاني في رسم إستراتيجيات وخطط التطوير المهني. |

| **Clusters and competencies** | **Stage 1** | **Stage 2** | **Stage 3** |
| --- | --- | --- | --- |
| 1. **Education, Training, and Development**   Supports the education, training, and development of self and others. Promotes a learning culture within the organization.   1. التعليم والتدريب والتطوير المستمر   *القدرة على دعم تعليم وتدريب و تطور الاّخرين ونشر ثقافة التعلم والتطوير المستمر ضمن مؤسسة العمل.* | | | |
| - 1. Links Practice and Education   ربط التعليم والتدريب بالممارسة المهنية | Participates **in the delivery** of didactic/experiential education and training.  أن يشارك الصيدلاني في تقديم برامج التعليم النظرية واالعملية**.** | Participates in **creation or development** of didactic/ experiential education and training.  أن يشارك الصيدلاني في تطوير برامج التعليم النظرية والعملية**.** | Shapes, contributes to /or is **accountable for the creation or development** of **higher education qualification**(s).  أن يكون الصيدلاني مشاركاً في تشكيل (أو مسؤولا عن تطوير) برامج التعليم العليا. |
| - 1. Educational Policy   السياسات التعليمية | Demonstrates an **understanding** of current educational policies relevant to workforce development.  أن يظهر الصيدلاني فهماً للسياسات التعليمية والتدريبية الحالية المتعلقة بالقوى العاملة الصيدلانية | Demonstrates ability to **interpret** national policy in order to design strategic approaches for local workforce education planning and development.  أن يظهر الصيدلاني قدرةً على تطوير استراتيجيات للتعليم والتدريب للقوى العاملة على المستوى المحلي بناء على فهمه وإدراكه للسياسات الوطنية. | Shapes and **contributes** to national education and workforce planning and development policy.  أن يشارك الصيدلاني في تخطيط ورسم السياسات الوطنية للتعليم والتدريب للقوى العاملة |

| 1. **Education, Training, and Development**   (See the page above) | **Evidence** | | | | | | | | | | | |
| --- | --- | --- | --- | --- | --- | --- | --- | --- | --- | --- | --- | --- |
|  | Member of, or provide  advice to, a local or  group or committee | Member of, or provide  advice to, a regional,  national or international  group or committee | Active Teaching role | Educational development,  design & provision | Active research  participation (includes  publications) | Research development & leadership (also includes publications) | Professional standing &  peer status | Documented expert  practice | Managing process &  leadership (local level) | Managing process &  Leadership (national  level) | Staff management | Any OTHER appropriate  Documentation |
| **5.1. Role Model** القدوة والنموذج  Stage 1 € Stage 2 € Stage 3 € | € | € | € | € | € | € | € | € | € | € | € | € |
| - 1. **Mentorship** الإرشاد والتوجيه   Stage 1 € Stage 2 € Stage 3 € | € | € | € | € | € | € | € | € | € | € | € | € |
| - 1. **Conducting, Education and Training**   التعليم والتدريب  Stage 1 € Stage 2 € Stage 3 € | € | € | € | € | € | € | € | € | € | € | € | € |
| - 1. **Professional Development**   التطوير المهني  Stage 1 € Stage 2 € Stage 3 € | € | € | € | € | € | € | € | € | € | € | € | € |
| - 1. **Links Practice and Education**   ربط مخرجات التعليم والتدريب بالممارسة المهنية  Stage 1 € Stage 2 € Stage 3 € | € | € | € | € | € | € | € | € | € | € | € | € |
| - 1. **Educational Policy**   السياسات التعليمية  Stage 1 € Stage 2 € Stage 3 € | € | € | € | € | € | € | € | € | € | € | € | € |

| **If any of the competencies was not achieved, explain why** |  |
| --- | --- |

| **Clusters and competencies** | **Stage 1** | **Stage 2** | **Stage 3** |
| --- | --- | --- | --- |
| 1. **Research and Evaluation**   Uses research to deliver effective practice. Identifies and undertakes research to inform practice   1. البحث والتقييم   *القدرة على تطوير الممارسة الصيدلانية والأداء الوظيفي بالإستناد على أساليب البحث العلمي* | | | |
| 6.1. Critical Evaluation  التحليل والتقييم الناقد | Demonstrates ability to critically evaluate and review the literature as well as **suggest changes to practice**.  أن يظهر الصيدلاني قدرةً على تقييم ومراجعة الأدلة العلمية بشكل ناقد وتحليلي واقتراح تغييرات مناسبة لمكان العمل. | Demonstrates ability to critically evaluate, and review the literature, to **apply evidence- based practice.**  أن يظهر الصيدلاني قدرةً على تقييم ومراجعة الأدلة العلمية بشكل ناقد وتحليلي لتطبيق الممارسة المبنية على الأدلة | undertakes **peer review** activities within practice.  أن يقوم الصيدلاني بأنشطة المراجعة الناقدة للآخرين في مجال العمل. |
| - 1. Develops and Evaluates Research   تطوير وتقييم البحث العلمي | Demonstrates ability to **formulate** appropriate and rigorous research questions based on identified gaps in the evidence-based practice  أن يتمكن الصيدلاني من صياغة أسئلة بحثية بشكل دقيق ومناسب بعد التعرف على وجود النقص في الأدلة العلمية . | Demonstrates ability to **design** rigorous protocol to address previously formulated research questions.  أن يظهر الصيدلاني قدرة على تصميم برنامج بحثي دقيق للإجابة على أسئلة بحثية تم تحديدها مسبقا. | Demonstrates ability to **lead** in the development and conduct of research  أن يظهر الصيدلاني قدرة على قيادة تطوير وإجراء البحث العلمي |
| - 1. Creates Evidence   تقديم الدليل العلمي | Demonstrates ability to generate new evidence suitable for presentation at **local level**.  أن يكون الصيدلاني قادراً على إنتاج أدلة علمية جديدة و تقديم نتائج الأبحاث العلمية على المستوى المحلي. | Demonstrates ability to generate new evidence suitable for presentation **at research or professional symposium**.  أن يكون الصيدلاني قادراً على إنتاج أدلة علمية جديدة وتقديم نتائج الأبحاث العلمية في المؤتمرات والندوات العلمية. | Demonstrates **authorship** of primary evidence and outcomes in **peer reviewed media**.  أن يقوم الصيدلاني بنشر نتائج الأبحاث العلمية في المجلات المحكمة علميا. |
| - 1. Applies Research Evidence into Working Practice   تطبيق نتائج البحث العلمي على الأداء المهني والوظيفي | Demonstrates ability to apply research and evidence-based practice into **own practice**.  أن يكون الصيدلاني قادراً تطبيق نتائج البحث العلمي في أدائه المهني والوظيفي. | Demonstrates ability to apply research and evidence-based practice **within the team and/or organization.**  أن يكون الصيدلاني قادراً تطبيق نتائج البحث العلمي في الأداء المهني والوظيفي لفريق العمل أو/مؤسسة العمل. | Is able to use research evidence to shape policy/procedure at an **organization and/or local, national, regional and international level**.  أن يكون الصيدلاني قادراً على إستخدام نتائج البحث العلمي في تشكيل سياسات وبرامج على مستوى مؤسسة العمل أو \والمستوى المحلي والوطني والإقليمي والعالمي. |
| **Clusters and competencies** | **Stage 1** | **Stage 2** | **Stage 3** |
| 1. **Research and Evaluation**   Uses research to deliver effective practice. Identifies and undertakes research to inform practice   1. **البحث والتقييم**   *القدرة على تطوير الممارسة الصيدلانية والأداء الوظيفي بالإستناد على أساليب البحث العلمي* | | | |
| - 1. Supervises Others Undertaking Research   الإشراف على برامج ومشاريع البحث العلمي للآخرين | Demonstrates understanding of the principles of research governance.  أن يكون الصيدلاني مدركاً للمعايير والمبادئ والقيم الناظمة للبحث العلمي. | Is able to contribute to research supervision in **collaboration with research expert**s.  أن يكون الصيدلاني قادرا على المساهمة في الإشراف على الأبحاث العلمية بالتعاون مع فريق من المختصين. | Is a research project **supervisor** **for others**  أن يكون الصيدلاني مشرفا على أبحاث الآخرين |
| - 1. Establishes Research Partnerships   إنشاء شراكات تعاون للبحث العلمي | Demonstrates ability to work as a **member** of the research team.  أن يظهر الصيدلاني قدرةً على المشاركة في مشروع بحث علمي ضمن فريق بحث. | Demonstrates ability to **establish multidisciplinary links** to conduct research projects.  أن يظهر الصيدلاني قدرة على إيجاد نقاط تواصل مع باحثين من مجالات متعددة للعمل على مشاريع وبرامج البحث العلمي. | Demonstrates ability to show **leadership within multidisciplinary** research teams concerning the conduct of research.  أن يظهر الصيدلاني قدرةً على قيادة وإدارة فريق من الباحثين من مجالات متعددةفي مشروع أو برنامج بحث علمي. |

| 1. **Research and Evaluation**   (See the page above) | **Evidence** | | | | | | | | | | | |
| --- | --- | --- | --- | --- | --- | --- | --- | --- | --- | --- | --- | --- |
|  | Member of, or provide  advice to, a local or Trust  group or committee | Member of, or provide  advice to, a regional,  national or international  group or committee | Active Teaching role | Educational development,  design & provision | Active research  participation (includes  publications) | Research development &  leadership (also includes  publications) | Professional standing &  peer status | Documented expert  practice | Managing process &  leadership (local level) | Managing process &  Leadership (national  level) | Staff management | Any OTHER appropriate  Documentation |
| **6.1. Critical Evaluation**  التحليل والتقييم الناقد  Stage 1 € Stage 2 € Stage 3 € | € | € | € | € | € | € | € | € | € | € | € | € |
| - 1. **Develops and Evaluates Research**   تطوير وتقييم برامج ومشاريع البحث العلمي  Stage 1 € Stage 2 € Stage 3 € | € | € | € | € | € | € | € | € | € | € | € | € |
| - 1. **Creates Evidence**   تقديم الدليل العلمي المثبت  Stage 1 € Stage 2 € Stage 3 € | € | € | € | € | € | € | € | € | € | € | € | € |
| - 1. **Applies Research Evidence into Working Practice**   تطبيق وعكس نتائج البحث العلمي على الأداء المهني والوظيفي  Stage 1 € Stage 2 € Stage 3 € | € | € | € | € | € | € | € | € | € | € | € | € |
| - 1. **Supervises Others Undertaking Research**   الإشراف ومتابعة برامج ومشاريع البحث العلمي  Stage 1 € Stage 2 € Stage 3 € | € | € | € | € | € | € | € | € | € | € | € | € |
| - 1. **Establishes Research Partnerships**   إنشاء شراكات تعاون للبحث العلمي  Stage 1 € Stage 2 € Stage 3 € | € | € | € | € | € | € | € | € | € | € | € | € |

| **If any of the competencies was not achieved, explain why** |  |
| --- | --- |

**Table 1: Examples for evidence per domain**

| **Competency** | **Evidence category** | - **Evidence examples** |
| --- | --- | --- |
| Expert Professional Practice | 1.Member of, or provide advice to, a local or group or committee | - Member in pharmacy or clinical committee - Communication of a pharmacy plan to hospital management e.g., presentations |
|  | 2. Member of, or provide advice to, a regional, national or international group or committee. | - Chair of national/regional/international specialist interest group - Guidelines on the use and monitoring of anticoagulants and your role in its development (e.g., perioperative management of anticoagulants) |
|  | 3. Active Teaching role | - Tutor/examiner for students |
|  | 4. Educational development, design & provision | - Lesson plans for teaching records of workshops /lectures /tutorials |
|  | 5. Active research participation (includes publications) | - Editor of reference book or chapters in specialist area - Published research papers - Undertakes research work |
|  | 6. Research development & leadership (also includes publications) | - Coordinator of research project/ward-based project - Peer reviewer for academic journal |
|  | 7. Professional standing & peer status | - Invited to speak at National/international conferences - Written reports prepared for directorate meeting and verbal presentation |
|  | 8. Documented expert practice | - Case logs of patients managed, clinical interventions, drug enquiries - Documented examples of management of medication problems in complex cases - Records of consultation requests for specialist advice from the organization - Apply ideas from the literature to patients in the specialist area - Documented examples from ward round discussions |
|  | 9. Managing process & leadership (local level) | - Development of treatment guidelines/protocols - Coordinator of development of protocols handbook/formulary/shared care protocols - Introduction of new services or new ways of working - Introduction of systems to reduce medication risk |
|  | 10. Managing process & leadership (national level) | - Development of national drug therapy/ adherence/ risk management guidelines - Contribution to a national strategy for pharmaceutical care - Development of standards of practice at national level |
|  | 11. Staff Management | - Feedback and notes from junior colleagues within the pharmacy team |

**Table 1: Examples for evidence per domain**

| **Competency** | **Evidence category** | - **Evidence examples** |
| --- | --- | --- |
| Collaborative working relationship | 1.Member of, or provide advice to, a local or group or committee | - Member of multidisciplinary protocol review panels |
|  | 2. Member of, or provide advice to, a regional, national or international group or committee. | - Member of a team that works with specialists from other specialties/hospital/organisations |
|  | 3. Active Teaching role | - Lesson plans or teaching records of workshops /lectures /tutorials |
|  | 4. Educational development, design & provision | - Development of education and training programmes |
|  | 5. Active research participation (includes publications) | - Editor of reference book or chapters in specialist area - Published research papers - Undertakes research work |
|  | 6. Research development & leadership (also includes publications) | - Leader/member of a project that involves a multidisciplinary team at local or national level |
|  | 7. Professional standing & peer status | - Collaboration with other teams e.g., doctors to initiate a new service or expand an existing one - Invited to speak at National/international conferences - Written reports prepared for directorate meeting and verbal presentation |
|  | 8. Documented expert practice | - Documentation on collaborative management of patients with the primary team - Documented examples of management of medication problems in complex cases - Communication/Defending of controversial information to multidisciplinary peers - Records of consultation requests for specialist advice from the organization |
|  | 9. Managing process & leadership (local level) | - Narrative on how you influenced other practitioners to change the practice - Introduction of new services or new ways of working |
|  | 10. Managing process & leadership (national level) | - Development of national drug therapy/adherence/ risk management guidelines |
|  | 11. Staff Management | - Feedback and notes from other professional outside the pharmacy team |

**Table 1: Examples for evidence per domain**

| **Competency** | **Evidence category** | - **Evidence examples** |
| --- | --- | --- |
| Leadership | 1.Member of, or provide advice to, a local or group or committee | - Member of the strategic planning committee |
|  | 2. Member of, or provide advice to, a regional, national or international group or committee. | - Member of a national/international strategic body |
|  | 3. Active Teaching role | - Lesson plans or teaching records of workshops /lectures /tutorials |
|  | 4. Educational development, design & provision | - Lead role in developing formal academic qualifications |
|  | 5. Active research participation (includes publications) | - Editor of reference book or chapters in specialist area - Published research papers - Undertakes research work |
|  | 6. Research development & leadership (also includes publications) | - Leader of a project that involves a multidisciplinary team at local or national level |
|  | 7. Professional standing & peer status | - Invited to speak at National/international conferences - Written reports prepared for directorate meeting and verbal presentation |
|  | 8. Documented expert practice | - Responsibility for approving new drugs usage - Implementation of trial data into local practice procedures and policy |
|  | 9. Managing process & leadership (local level) | - Narrative on how you influenced other practitioners to change the practice - Coordinator of development of protocols handbook/formulary/shared care protocols |
|  | 10. Managing process & leadership (national level) | - Leader of the development and adoption of new policy or procedure at national level |
|  | 11. Staff Management | - Lead role in guiding the pharmacy team through service reconfigurement /staffing changes |

**Table 1: Examples for evidence per domain**

| **Competency** | **Evidence category** | - **Evidence examples** |
| --- | --- | --- |
| Management | 1.Member of, or provide advice to, a local or group or committee | - Your participation in creating a new policy on managing anticoagulation in the hospital anticoagulation services |
|  | 2. Member of, or provide advice to, a regional, national or international group or committee. |  |
|  | 3. Active Teaching role | - Lesson plans or teaching records of workshops /lectures /tutorials |
|  | 4. Educational development, design & provision | - Development of education and training programmes |
|  | 5. Active research participation (includes publications) | - Editor of reference book or chapters in specialist area - Published research papers - Undertakes research work |
|  | 6. Research development & leadership (also includes publications) | - Leader of a project that involves a multidisciplinary team at local or national level |
|  | 7. Professional standing & peer status | - Developing/supervising policies and procedures (e.g., for the inpatient pharmacy) - Invited to speak at National/international conferences - Written reports prepared for directorate meeting and verbal presentation |
|  | 8. Documented expert practice | - Responsibility for approving new drugs usage - Implementation of trial data into local practice procedures and policy |
|  | 9. Managing process & leadership (local level) | - Contribution to a local strategy for pharmaceutical care - Development of business plan for service - Introduction of new services or new ways of working - Introduction of systems to reduce medication risk |
|  | 10. Managing process & leadership (national level) | - Development of national risk management guidelines - Development of standards of practice at national level |
|  | 11. Staff Management | - Developing/facilitating resourcing for staff - Managing and guiding the pharmacy team through service re-configurement /staffing changes - Performance appraisal for staff |

**Table 1: Examples for evidence per domain**

| **Competency** | **Evidence category** | - **Evidence examples** |
| --- | --- | --- |
| Education, training and development | 1.Member of, or provide advice to, a local or group or committee | - Presenting patients’ feedback/ satisfaction with services (e.g., anticoagulation patient education service) |
|  | 2. Member of, or provide advice to, a regional, national or international group or committee. | - Member of internal CPD committee |
|  | 3. Active Teaching role | - Continuing education programs on anticoagulation conducted to help bridge knowledge gaps and to share clinical pearls - Teaching at a national symposium |
|  | 4. Educational development, design & provision | - Training designed and conducted for staff involved in the inpatient anticoagulation service |
|  | 5. Active research participation (includes publications) | - Editor of reference book or chapters in specialist area - Published research papers - Undertakes research work |
|  | 6. Research development & leadership (also includes publications) | - Peer reviewer for academic journal |
|  | 7. Professional standing & peer status | - Invited to speak at National/international conferences - Written reports prepared for directorate meeting and verbal presentation |
|  | 8. Documented expert practice | - CPD facilitator for pharmacy team - Apply ideas from the literature to patients in the specialist area |
|  | 9. Managing process & leadership (local level) | - Coordinator of development of protocols handbook/formulary/shared care protocols |
|  | 10. Managing process & leadership (national level) | - Development of national education and training guidelines - Development of standards of education at national level |
|  | 11. Staff Management | - CPD facilitator for pharmacy team |

**Table 1: Examples for evidence per domain**

| **Competency** | **Evidence category** | - **Evidence examples** |
| --- | --- | --- |
| Research | 1.Member of, or provide advice to, a local or group or committee | - Member of directorate/ ethics committee |
|  | 2. Member of, or provide advice to, a regional, national or international group or committee. | - Provide advice to professional/ strategy bodies |
|  | 3. Active Teaching role | - Lesson plans or teaching records of workshops /lectures /tutorials |
|  | 4. Educational development, design & provision | - Development of education and training programmes |
|  | 5. Active research participation (includes publications) | - Quality audit on the service |
|  | 6. Research development & leadership (also includes publications) | - Publication of results - Reviews of research protocols at local or national level |
|  | 7. Professional standing & peer status | - Invited to speak at National/international conferences - Written reports prepared for directorate meeting and verbal presentation |
|  | 8. Documented expert practice | - Apply ideas from the literature to patients in the specialist area |
|  | 9. Managing process & leadership (local level) | - Supervisor of medical or/and pharmacy student projects |
|  | 10. Managing process & leadership (national level) | - Conducted critical review of literature to develop national practice guidelines - Documented national audits of prescribing quality |
|  | 11. Staff Management | - Quality audit on staff performance |
